# Microstructure, Mechanical Properties, In Vitro Biodegradability, and Biocompatibility of Mg-Zn/HA Composites for Biomedical Implant Applications

**DOI:** 10.3390/ma16165669

**Published:** 2023-08-17

**Authors:** Wei Lu, Yinling Zhang, Taolei Wang

**Affiliations:** 1 School of Mechanical Engineering, Suzhou University of Science and Technology, Suzhou 215009, China; 2 School of Material Science and Engineering, Tongji University, Shanghai 201209, China; weilu@tongji.edu.cn; 3 School of Mechanical Engineering, Shandong University of Technology, Zibo 255049, China; yinling@sdut.edu.cn

**Keywords:** magnesium alloys, PM, hydroxyapatite, bone implants

## Abstract

Recently, Mg-Zn/hydroxyapatite (HA) composites have attracted much attention as potential candidates for use in bone implants. In this paper, the MgZn/HA composites were prepared using powder metallurgy (PM) and the merging mechanism of MgZn and HA particles was investigated by adjusting the weight ratio of the HA powder. The evolution of the HA distribution in the matrix was examined using SEM and micro-CT images. Afterward, the mechanical properties and biocompatibility of the composites were discussed in detail. The results revealed that the mechanical properties and biocompatibility of the Mg-Zn/HA composites were significantly affected by the HA content. Composites with a low HA content showed increased porosity, improved mechanical strength, and enhanced corrosion resistance after ball milling and cold pressing. These results underscore the importance of optimizing the HA content in Mg-Zn/HA composites for bone implants. Based on our findings, PM Mg-Zn/HA composites with a moderate HA content demonstrate the most promising characteristics as bone implants. The insights gained from this work contribute to the advancement of bone implant materials and hold great potential for enhancing orthopedic surgery outcomes.

## 1. Introduction

Over the past decade, magnesium and its alloys have gained much attention in the field of biomaterials [1] because of their biodegradable nature [2] and high strength-to-density ratio [3]. However, up to now, clinical applications of magnesium-based implants have been relatively scarce due to their fast and uncontrollable degradation rate in physiological solutions for the human body [4], leading to harmful hydrogen gas pockets, premature loss of mechanical strength, and local increases in alkalinity [5].

Addressing the poor corrosion resistance of magnesium and its alloys is the most appropriate strategy since a low corrosion rate of Mg-based implants implies a decrease in the extent of hydrogen evolution and alkalization [6], which would enable the human body to absorb or consume the corrosion products gradually. One practical approach is fabricating protective coatings with multi-functionalities on the magnesium substrate [7]. In recent years, several techniques for converting or depositing coatings onto Mg substrates have been explored, including chemical conversion [8], plasma electrolytic oxidation (PEO) [9], micro-arc oxidation [10], hydrothermal treatment [11], and immersion coating [12]. However, the functionality of surface coatings is limited to the early stages of immersion in physiological solutions, as only the exposed surfaces of implants can be coated, leaving the entire substrate untreated [13]. Moreover, under mechanical loading, most coatings are prone to delamination [14].

Thus, improving the corrosion resistance of a substrate is of paramount importance. Techniques such as alloying [15], rapid cooling [16], and introducing deformation processing methods like equal-channel angular pressing [17] and melt processing with ultrasound treatment [18] have been shown to significantly impact corrosion resistance and enhance mechanical properties by obtaining fine-grained structures. Additionally, metal matrix composites (MMCs) present a potential solution that can be to improve both the mechanical properties and corrosion rate of magnesium-based materials [19,20]. By incorporating reinforcement materials with good biodegradability and biocompatibility, the mechanical properties and corrosion resistance of magnesium-based composites can be tailored. For instance, ceramic [21] or diamond nanoparticles [18] can impart additional benefits to materials and should be further investigated in the scientific literature to determine their advantages.

The preparation of magnesium-based composites requires reinforcement materials with excellent biodegradability and biocompatibility in order to enhance the composite’s corrosion resistance [22] and mechanical strength [23]. Among the available biomedical materials, hydroxyapatite (Ca_10_(PO_4_)_6_(OH)_2_, HA) has been widely utilized [24] due to its excellent biocompatibility and bioactivity as its chemical and structural properties resemble those of bone and tooth minerals [25]. On one hand, the presence of CaP ceramic particles or scaffolds can promote the formation of a passive film on the composite material’s surface, retarding the corrosion rate and exhibiting a more uniform corrosion behavior [25]. However, it should be noted that in composites, particularly during the early stages of corrosion, galvanic corrosion occurs rapidly at the interface between the substrate and ceramic particles, resulting in deeper and more severe corrosion due to the infiltration of the corrosive solution into the composite material [26,27].

To further enhance the corrosion resistance and mechanical properties of magnesium-based composites, researchers have explored the effect of nanoparticle additives. In particular, studies have examined how the content of HA affects the performance of composites [28,29], including the degradation rate and mechanical strength. These studies have shown promising results in terms of improving the overall properties of the composites. However, to better understand the implications of such additives and their specific effects on corrosion resistance, more in-depth studies are warranted. Consequently, further research is needed to fully explore and validate the advantages of these techniques for enhancing the corrosion resistance of magnesium-based composites.

Therefore, this research aims to improve the corrosion resistance, mechanical properties, and biocompatibility of magnesium-based composites by incorporating HA as a reinforcement material. A Mg alloy matrix with 6 wt.% Zn was employed to improve the mechanical properties and biocompatibility of the matrix, as demonstrated in the previous literature [30]. This investigation focuses on tailoring the HA content to optimize the performance of the Mg-Zn/HA composites, ultimately contributing to the development of biodegradable implants with enhanced functionality and performance in orthopedic applications.

## 2. Materials and Methods

### 2.1. Preparation of Mg-Zn/HA Composites

Pure magnesium powders (Mg, 99.9% purity, sphere, 1000 mesh, Institute of Metallurgy, Shijiazhuang, Hebei, China), zinc powders (Zn, 99.9% purity, sphere, 1000 mesh, Institute of Metallurgy, Shijiazhuang, Hebei, China), and nano-HA (99.9% purity, average particle size about 60 nm, needle-like, Emperor Nano, Nanjing, China) were used as raw materials to prepare Mg-Zn/HA composites. Six different mixtures with certain HA content were mixed as Mg-Zn-0HA, Mg-Zn-5HA, Mg-Zn-10HA, Mg-Zn-15HA, Mg-Zn-20HA, and Mg-Zn-25 HA (wt.%), named MZH0, MZH5, MZH10, MZH15, MZH20, and MZH25, respectively. The mixtures were transferred into a stainless-steel mold. Uniaxial pressing was applied at a pressure of 100 MPa for 10 min, followed by sintering at a temperature of 500 °C for 2 h in a vacuum furnace (<10^−4^ Pa).

### 2.2. Characterization of Mg-Zn/HA Composites

The structure and phase of the Mg-Zn/HA composites were examined using X-ray diffraction (XRD) with Cu Kα radiation and field emission scanning electron microscopy (FE-SEM) (Supra 40, Zeiss, Jena, Germany) equipped with EDS.

High-resolution X-ray micro-tomography (micro-CT) was used to characterize the pores and defects of the sample, and a Bruker 3D X-ray SkyScan 1275 (Bremen, Germany) was used to reconstruct the internal structural details of the sample. Due to the size limitation of this X-ray machine, a cutting machine was used to cut a part of the original sample with a size of 2 cm × 2 cm × 2 cm. After the sample was installed in the scanner, 40 kV X-rays were used to scan the sample from different angles. Then, NRecon software (v.1.7.4.2) was used to reconstruct the scan to obtain the complete 3D structural details of the sample.

The porosities of samples were measured using Archimedes’ principle. The samples were cut into pieces with a diameter of 10 mm and a thickness of 20 mm. The dimensions of the samples were measured using a Vernier caliper to produce a total volume (V). The samples were first weighed using an electronic analytical balance; the weight was recorded as m_0_. Then, the sample was dipped into deionized water and suspended from an analytical balance to obtain wet weight (m_1_). All weights were in grams, and ρ was the density of deionized water (1 g/cm^3^). Thus, the porosity (θ) of the sample was calculated using the following equation:

θ = 100% × (1 − (m_1_ − m_0_)/(ρ × V)) (1)

The Vickers micro-hardness of the bulk samples was measured using a micro-hardness tester by applying a load of 300 g onto the polished surfaces of the samples. For each sample, 10 separate indents were created on the investigated surface.

### 2.3. Immersion Tests

Immersion tests were carried out in simulated body fluid (SBF) (Hank’s balanced salts, H2387, Sigma-Aldrich, Seoul, Republic of Korea) at 37 °C for 20 days. The volume of SBF was determined according to the mass of the sample (1 g sample/10 mL SBF solution). ZK60 substrate polished with 2000# SiC paper was used as the control group. After certain hours, the samples were rinsed with deionized water, dried in nitrogen flow, and weighed. The pH values of the immersion solution were measured using a pH meter (PHSJ-6L, Leici, Shanghai, China). H2 release was measured using the displacement method.

### 2.4. Cell Culture

Four-week-old male SPF SD rats were provided by the experimental animal center of Shanghai No.9 People’s Hospital. Rats were sacrificed via neck dislocation; bone marrow was obtained by rising the bone marrow cavity with DMEM after cutting off the epiphysis at both ends of the bone. The collected bone marrow washing fluid was centrifuged at 1800 rpm for 10 min. The cell suspension was seeded into a culture dish. The culture conditions were 37 °C and 5% CO_2_, and passage 2 to passage 4 of bone marrow mesenchymal stem cells were used in this research.

### 2.5. Alkaline Phosphatase (ALP) Activity

Mesenchymal stem cells (MSCs) were seeded onto the samples in 24-well plates at a density of 3 × 10^4^ cells/well and cultured for 7 days in an osteogenic medium. Parallel sets of cells were cultured for 7 days with a mixture of 50% osteogenic induction medium and 50% culture medium (CM) of materials. As controls, cells were incubated only in osteogenic induction medium. CM was replaced at day 3 with an equal volume of fresh medium. ALP activity was assessed in cell layers by determining the release of p-nitrophenol from p-nitrophenylphosphate (Sigma, St. Louis, MO, USA) at 37 °C and a pH of 10.5. The data were normalized to the total protein amounts in cell layers determined using the Bio-Rad (Hercules, CA, USA) protein assay.

### 2.6. Statistical Analysis

Results were averaged and expressed as the mean ± standard deviation. Statistical analysis was performed using ANOVA. A value of *p* < 0.05 was considered statistically significant.

## 3. Results

### 3.1. Microstructure (XRD/SEM/EDS/CT)

**Figure 1:**
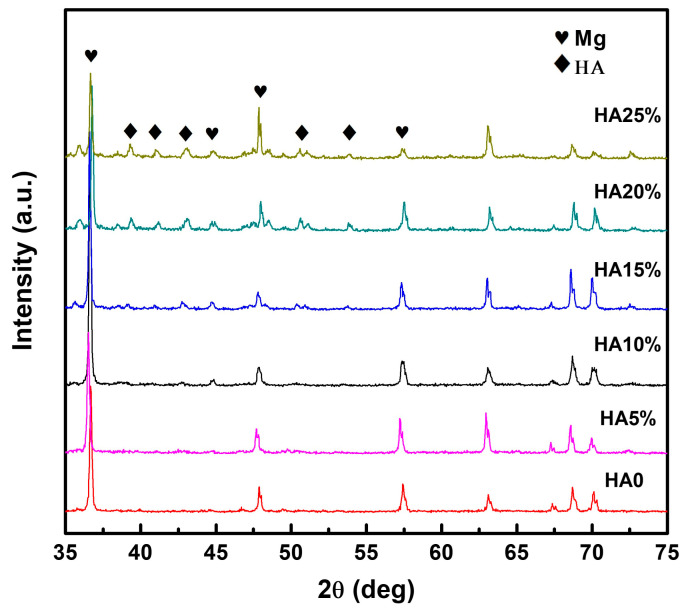
XRD patterns of powder-metallurgy Mg-Zn composite samples with different amounts of HA reinforcement.

The XRD patterns of powder-metallurgy Mg-Zn composite samples with different amounts of HA reinforcement are shown in Figure 1. The diffraction peaks of Mg are sharp and intense in all samples, indicating their highly crystalline nature. No characteristic diffraction peaks of Zn are observed due to its lower loading content and good dispersion in the Mg matrix. When the content of HA is less than 15%, no prominent diffraction peaks from HA can be identified from the XRD patterns. The weak diffraction peaks indexed as HA are observed in the XRD patterns of the composite samples with HA content higher than 15%. Other than these, no obvious peaks can be found, indicating that no chemical reactions between the Mg-Zn alloy and HA occurred during the milling process.

**Figure 2:**
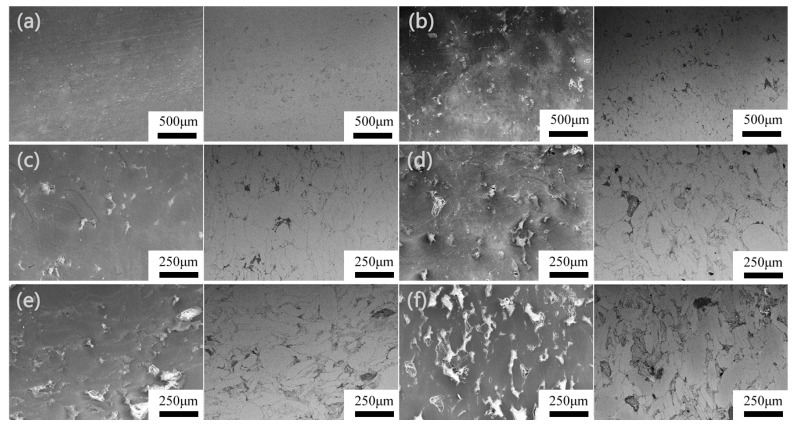
SEM images of the surface of Mg-Zn/HA composites, (**a**) (as-casted Mg-Zn alloys), (**b**) (5 wt.% HA), (**c**) (10 wt.% HA), (**d**) (15 wt.% HA), (**e**) (20 wt.% HA), (**f**) (25 wt.% HA).

Figure 2 shows the scanning electron microscopy images of the sintered Mg-Zn/HA composites, with the secondary electron images on the left and the corresponding backscattered electron images on the right. As can be seen from Figure 2a, the sintered Mg-Zn sample contained a small number of pores with pore sizes of less than 1 μm, indicating that the sintering parameters used in the present study were good enough to obtain a nearly compact structure. In contrast, the microstructures of the Mg-Zn/HA composites were composed of two phases, i.e., a dark background and continuously distributed white particles (Figure 2b–f), which is consistent with the results obtained from the XRD analysis (Figure 1). It was clear that the dark background and white particles corresponded to the Mg and HA phases, respectively. It was also observed that the HA phase was uniformly distributed as individual particles at the grain boundary of the Mg-Zn matrix. Part of this phase also formed interconnected clusters distributed in the matrix when the content of HA was less than 15% in the Mg-Zn/HA composites. The Mg matrix and HA reinforcement particles appeared well combined at the interface. When the content of HA increased from 15% to 25% (Figure 2d–f), HA particles gradually formed continuous networks at the grain boundary throughout the Mg matrix. These networks were composed of agglomerates of HA particles and coarsened with increasing content of HA in the Mg-Zn/HA composites.

**Figure 3:**
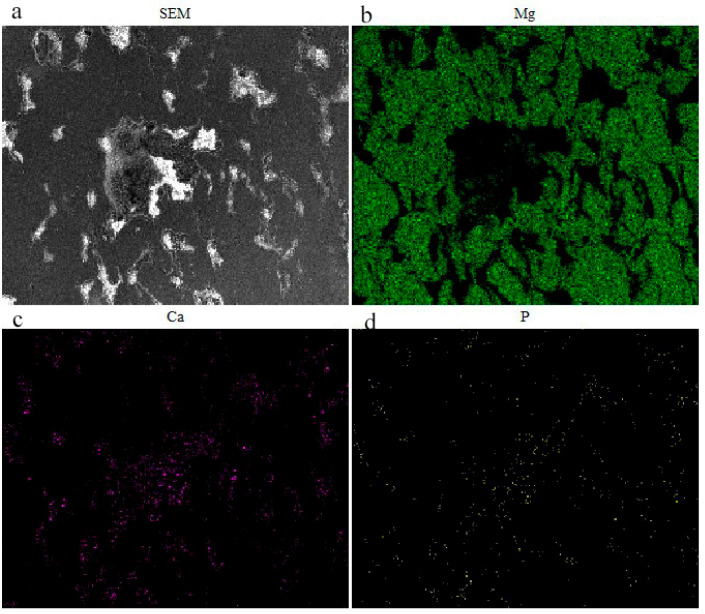
SEM image of Mg-Zn/15 wt.% HA composite (**a**) and element distribution images of Mg (**b**), Ca (**c**) and P (**d**).

The SEM microstructure of the interface between HA and the Mg-Zn alloy and the corresponding EDS maps for Mg, Ca, and P elements are shown in Figure 3. It can be seen from Figure 3a that no noticeable gap and discernible debonding layer can be found in the interface between Mg-Zn grains and HA particles. Generally, the HA phase and the Mg-Zn alloy can be distinguished in Figure 3b–d, in which the HA phase reveals the highest Ca and P concentrations and the Mg phase reveals the highest Mg concentration. Although the distribution of Mg and Ca crosslinked, indicating that some molten Mg alloys could penetrate the HA cluster, apparent separation could still be observed in the Mg, especially in the central part where the vast HA cluster occurred, indicating that the large HA cluster resulted in an insufficient merging between the MgZn particles.

**Figure 4:**
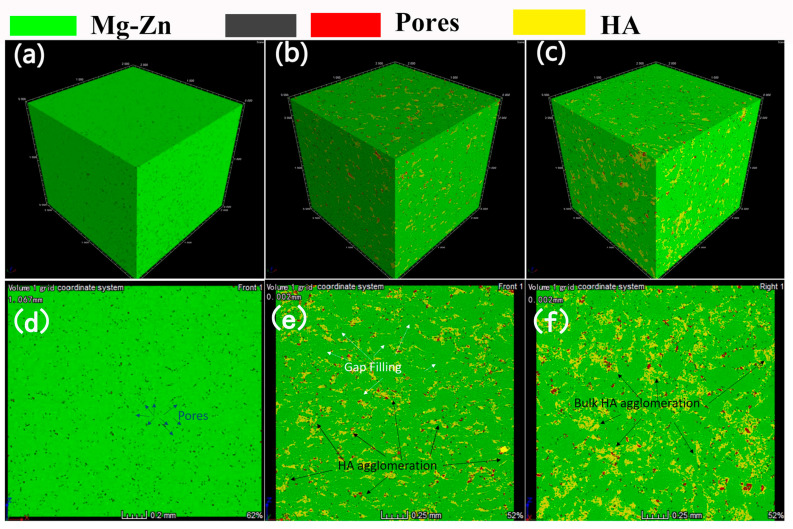
Micro-CT scan of the pressed Mg-Zn/HA samples, (**a**) (0 wt.% HA), (**b**) (10 wt.% HA), (**c**) (25 wt.% HA), and cross-section CT images of Mg-Zn/HA samples (**d**) (0 wt.% HA), (**e**) (10 wt.% HA), (**f**) (25 wt.% HA).

Figure 4 shows the micro-CT images of the sintered samples. In these images, the green parts represent the Mg-Zn phase, the yellow pieces represent the HA particles, and the black features represent the pores. In the MZH0 sample, it can be observed that the pores were uniformly distributed in the pure Mg-Zn matrix. With the small addition of HA particles in the MZH15 samples, both the size and number of the pores were reduced, indicating that the HA particles filled a portion of the pores between the Mg-Zn grains. Other than the HA particles filling the voids, some small HA agglomerations can be observed. As the HA content kept increasing, the agglomeration of HA was aggravated, and a large amount of bulk HA agglomeration can be observed in the micro-CT images of MZH25 (Figure 4f), along with increased red parts representing the incomplete integration of the Mg-Zn and HA phases.

The porosities of the sintered samples (Table 1) were calculated using Archimedes’ principle. As shown in Table 1, the porosity of MZH0 was 3.9%, due to the formation of minimal cavities via insufficient compaction [31]. With the addition of HA, part of the minimal cavities filled the HA particles, the porosity decreased, and the porosity of MZH15 decreased to 3.0%. According to the Andreasen equation [32], the expression of the particle size distribution of powder particles is:

(2) $$W=AD^{q}$$

where *W* is the mass fraction of particles with a particle size smaller than *D*, and *A* and *q* are the number of empirical rows to adapt to the adjustment of the particle size distribution. The loose density value reaches its largest when *q* is between 0.5–0.67. In this experiment, an appropriate increase in the proportion of tiny particles of HA helps to fill the gaps between large Mg-Zn particles. When the HA content is less than 15 wt.%, the Mg-Zn phase serves as the skeleton and the nano-HA particles fill the voids between the Mg-Zn degrees. At the same time, the ball milling process endows the uniform distribution of the HA particles around the Mg-Zn particles [33]. Therefore, the porosity of the sample reached the lowest value when the HA content was 15 wt.%, and the HA showed a uniform distribution around the Mg-Zn phase.

However, some HA clusters occurred, especially when the HA content was relatively high. Due to the inferior plastic deformation ability and high sintering temperature (above 1000 °C) of HA, the reduction in the number of pores between the HA particles using the cold-pressing and sintering processes was relatively small. Furthermore, excessive HA addition caused more HA clusters of bigger sizes, which can be seen in MZH25. In addition, some bulk HA clusters inhibited the merging of the Mg-Zn particles. Therefore, even though part of the HA particles filled the voids between the Mg-Zn powders, excessive HA addition (≥20%) still increased the porosity of the composites.

### 3.2. Mechanical Properties (Hardness/Compressive Strength/Elastic Modulus/Immersion Time Dependence)

Table 2 shows the mechanical strength of the as-sintered Mg-Zn/HA composites. As shown in Table 2, it is clear that the addition of HA deteriorates the plasticity of the Mg-Zn/HA composites. The elastic modulus of the Mg-Zn alloy was 39.2 GPa, while that of the MZH25 was 29.8. The decreased elastic modulus with the addition of HA should be subjected to the HA particles around the Mg-Zn grain boundary, suppressing the deformation and movement of Mg-Zn grains. In the meantime, the hardness and compressive strength showed a different trend owing to the grain refinement via HA addition. Since HA particles are still stable at high temperatures, the growth of Mg-Zn particles during the sintering process is affected by adding HA particles and pores. The experience size is calculated according to Zenar’s Equation (4) [34]:

(3) $$R=\frac{4r}{3f}$$

where *R* is the average radius of the Mg-Zn grains; *r* is the average radius of the second phase (HA particles or pores); and *f* is the volume fraction of the second phase. The value of r is proportional to the value of *r*/*f*, which means that small HA particles with a large amount suppress the grain growth of the Mg-Zn phase, resulting in a fine-grained structure. Thus, when the HA content was 15 wt.%, the HA particles were uniformly distributed around the Mg-Zn stage, the *r*/*f* value was the lowest, and the composite had a fine-grained structure [35]. Grain refinement is one of the most effective ways of strengthening hexagonal close-packed magnesium [36]. Thus, the MZH15 sample had the highest hardness and compressive strength, which could be attributed to the grain refinement [37].

In addition, according to the Orowan mechanism [38], when a composite contains porosity or voids, the dislocations within the matrix material tend to interact with these defects. As reported in the literature [39], the presence of porosity creates stress concentrations around the voids, making it energetically favorable for dislocations to bypass or shear around the voids instead of propagating through them. This reduces the effective dislocation mobility, making it more difficult for dislocations to move and leading to an increase in the material’s yield strength. However, if the porosity is significant [40], it may weaken the bonding between the particles and the matrix, resulting in decreased strength and stiffness of the composite. This is consistent with the variation in the porosity and mechanical properties of the sample.

### 3.3. In Vitro Biodegradation (Weight Loss/H2 Evolution/pH)

**Figure 5:**
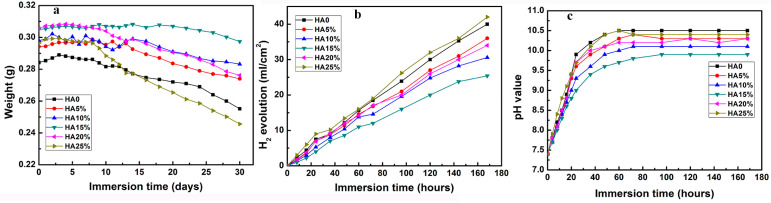
(**a**) Weight loss rates of the as-cast Mg-Zn alloy and Mg-Zn/HA composites, (**b**) hydrogen evolution, and (**c**) pH variation curves after 180 h of immersion in Hanks’ solution.

Figure 5 shows the weight of the sample (a), hydrogen evolution (b), and pH (c) of the SBF solution measured during the immersion of the samples for 170 h. The degradation of the Mg-Zn matrix during the immersion tests could be concluded as the following equations [41]:

Mg − 2e^−^→Mg^2+^ (4)

Zn − 2e^−^→Zn^2+^ (5)

H_2_O + 2e^−^→H_2_ + OH^−^ (6)

This reaction would lead to rapid weight loss, hydrogen release, and increased pH value. As can be seen from the figures, the as-casted Mg-Zn alloy showed rapid weight loss, extended hydrogen release, and fast pH value increase during the immersion [42]. HA addition of less than 15 wt.% improved the corrosion resistance of the composites, which could be attributed to three factors. Firstly, smaller HA particles with a larger surface area can act as cathodic sites, promoting localized corrosion, especially at grain boundaries, and leading to accelerated corrosion rates [43]. Secondly, a more uniform distribution of HA particles may hinder corrosive attacks and reduce the corrosion susceptibility of the composites [21]. Additionally, the presence of HA within the Mg matrix is helpful for the accumulation of Ca^2+^ ions on the Mg surface since HA can act as the nucleation site for the precipitation and growth of Ca^2+^ [44]. Similar to other research [45], the evolution of corrosion resistance with HA addition is almost similar to that of the porosity, and MZH15 showed the highest corrosion resistance.

### 3.4. Biocompatibility

**Figure 6:**
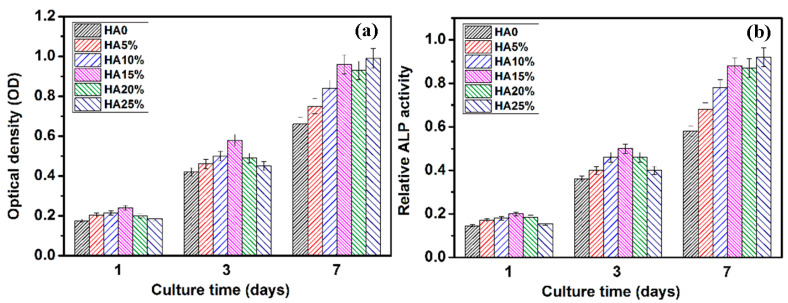
Optical density value (**a**) and relative ALP activity (**b**) of BMSCs after incubation with the as-casted Mg-Zn alloy and Mg-Zn/HA composites for 1, 3, and 7 days.

MTT assays were performed at 1, 3, and 7 days to evaluate the viability of BMSCs (bone marrow stromal cells). The cell density (proliferation and differentiation) of BMSCs cultured on the samples was then determined. As shown in Figure 6, the trend of the optical density was consistent with the corrosion resistance trend of the samples. MZH15 had the highest optical density among the six samples. It is reported that the rapid degradation of magnesium induces apoptosis [5]. HA addition can enhance the corrosion resistance and provide a relatively stable environment with a lower pH value for the cells to grow in [46]. Meanwhile, hydrogen bubbles are not released during the degradation of HA particles, and HA particles have higher specific surface area and roughness, which is more favorable for cell adhesion growth.

The differentiation of osteoblast bone cells can be evaluated by using ALP activity as a marker of osteoblastic activity [47]. Figure 6b shows that the relative ALP activity of the composites had the same trend as the optical density. It should be noted that HA can absorb more proteins such as fibronectin and vitronectin from the serum, promoting good binding with integrins. As a result, the osteoconductivity of samples with more HA nanoparticles improved. In addition, the addition of HA introduced more pores and cracks. This increased the roughness of the composites, which was a more favorable environment for cells to grow in. Therefore, after 7 days of incubation, the MZH25 with the highest HA content had the most increased ALP activity among these samples.

## 4. Conclusions

In conclusion, Mg-Zn/HA composites were prepared using ball milling and powder metallurgy methods. At a lower HA content (≤15 wt.%), the distribution of HA nanoparticles around Mg-Zn particles effectively reduces porosity and enhances mechanical properties and corrosion resistance. However, excessive HA content leads to the formation of HA clusters, negatively affecting mechanical properties and corrosion resistance. The structural features influenced by HA content play a crucial role in achieving desirable material properties, making these composites potential candidates for bone implants. Subsequent cell culture experiments demonstrated that HA addition promoted biological activity and osteoblast growth. Overall, understanding and controlling the structural effects of HA in Mg-Zn composites is key to harnessing their desired properties for biomedical applications.

## Figures and Tables

**Table 1:** Porosities of the pressed samples with different amounts of HA.

HA Content	Porosity (%)
0	3.9
5	3.5
10	3.2
15	3.0
20	3.6
25	4.2

**Table 2:** Mechanical properties of the samples with different additions of HA.

HA Content	HV	Compressive Strength (MPa)	Elastic Modulus (GPA)
0	91	253	39.2
5	106	274	37.5
10	123	292	34.8
15	136	306	32.4
20	126	295	31.3
25	115	280	29.8

## Data Availability

Not applicable.
